# Fall Prevalence and Associated Risk Factors Among the Elderly Population in Tabuk City, Saudi Arabia: A Cross-Sectional Study 2023

**DOI:** 10.7759/cureus.45317

**Published:** 2023-09-15

**Authors:** Abdallalh Alanazi, Safa Salih

**Affiliations:** 1 Preventive Medicine Department, Public Health Administration, Tabuk, SAU

**Keywords:** saudi arabia, prevention, risk factors, prevalence, elderly, falls

## Abstract

Background

Falls are common among older adults, and they constitute a major public health issue with high morbidity and mortality.

Aim

This study aimed to estimate the prevalence of falls and investigate the contributing risk factors among the elderly population in Tabuk City, Saudi Arabia.

Methods

This cross-sectional study recruited a random representative sample of the elderly aged ≥ 60 years. We collected data by interviewing the participants using a structured, Arabic-language questionnaire. It included personal information, a history of falls in the past three and 12 months, comorbidities, and environmental factors. The main outcome was a history of falls in the preceding year. Multivariable logistic regression was used to evaluate the association between potential risk factors and falls.

Results

The study included 296 participants. Most participants were female (66.9%), aged 60-69 years (68.2%), and married (68.9%). The self-reported prevalence of falls over the preceding 12 months was 25.3% (95% confidence interval (CI): 20.6-30.5). Older people with depressive symptoms had significantly increased vulnerability to falls (adjusted odds ratio (AOR): 0.452, 95% CI: 0.239-0.854). Environmental factors were associated with a 1.799 times (95% CI: 1.041-3.109) increased likelihood of fall, and gait impairment was the strongest risk factor (AOR: 2.775, 95% CI: 1.558-4.942).

Conclusions

Falls are common among the elderly population in Tabuk City, Saudi Arabia. Gait impairment, the presence of depressive symptoms, and environmental hazards were substantially associated with falls, suggesting that most falls are preventable.

## Introduction

The World Health Organization (WHO) identified a fall as an event enforcing a person to rest inadvertently on the ground, floor, or other lower level [1]. Falls are most prevalent (34-57.7%) among older adults (aged 60 years or more), and they constitute a worldwide major public health concern [2,3]. Falls are associated with high morbidity and mortality. Various hazards related to falls have been recorded, such as disability, loss of independence, functional decline, and greater fear of falling [4,5].

An increasing interest has recently been noticed regarding the problem of elderly falls and their consequences because of the increasing life expectancy and, subsequently, the proportion of the elderly population [6].

Several studies from developed countries have explored the burden of falls among the elderly [7-12]. Nevertheless, corresponding research addressing this problem in the Middle East is deficient. In Saudi Arabia, few studies have assessed falls among the elderly. Almegbel et al. [13] have found that the annual prevalence in Riyadh was 49.9%, while Alshammari et al. [14] have reported that 57.7% of elderly participants in Riyadh had a history of falls. A more recent study revealed an annual prevalence rate of 31.6% in Qassim province, Saudi Arabia [15].

Older individuals with a lack of physical activity and the presence of comorbidities such as hypertension and diabetes mellitus are more vulnerable to falls [16]. Polypharmacy, excessive alcohol consumption, and environmental risks, including poor interior design, insufficient lighting, and uneven flooring or surfaces, have been suggested as modifiable risk factors for falls [17]. Socioeconomic factors, such as low education levels, unemployment, and low income, might also increase the tendency to fall [16]. A history of previous falls has been identified as a risk factor of considerable importance for falls [18]. The recurrence of falls could be attributed to diminished physical fitness, postural instability, and inappropriate environmental conditions [19].

The reported burden and risk factors of falls among the elderly exhibit a wide variation, which is attributed to the differences in environmental factors and population-based characteristics [20]. Unfortunately, evidence is lacking on the true picture of this problem among the elderly in developing countries, including Saudi Arabia [15].

Falls and their consequent complications pose a major burden for the elderly, their caregivers, and the healthcare system [12]. Determining the prevalence and risk factors of falls among the elderly is essential to raising public awareness and helping healthcare authorities develop effective prevention strategies. Indeed, this could help reduce fall-related morbidity and mortality and enhance the quality of life among the elderly population.

Therefore, this study aimed to estimate the prevalence of self-reported falls and explore the related risk factors among the elderly population in Tabuk City, Saudi Arabia.

## Materials and methods

Study design, setting, and duration

This is a cross-sectional study conducted at ten primary healthcare centers in Tabuk City, Saudi Arabia, from the start of June 2023 to the end of August 2023.

Ethical considerations

This study obtained ethical approval from the Tabuk Institutional Review Board (approval number: TU-077/023/201). We asked the willing subjects to sign an informed written consent form before participating in the study. We informed the participants about the study objectives and methodology and that we would use their anonymized data only for research purposes while maintaining their confidentiality.

Sample size calculation and sampling technique

The sample size was estimated with an online sample size calculator (Raosoft, http://www.raosoft.com/samplesize.html) using a margin of error of 5%, a confidence interval of 95%, and an average expected prevalence of nearly 26.5% [21], depending on an average population size of nearly 25,000 elderly people in Tabuk City [22]. The minimum required sample size was 296 participants. We used a simple random sampling technique to recruit the study participants. We randomly chose 10 out of the 23 primary healthcare centers in Tabuk City, and we recruited participants from each center via a simple random sampling technique.

Eligibility criteria

The study included male and female elderly subjects who were aged 60 years or more and visited a primary healthcare center in Tabuk City, Saudi Arabia. Subjects who refused to participate, participants less than 60 years old, and those with incomplete data were excluded.

Data collection procedure

After obtaining ethical approval, we collected the participants’ data between June 11, 2023, and July 6, 2023, by filling out a structured, Arabic-language questionnaire via a direct interview with the participants during their visits to the primary health centers.

Data collection tool

We developed the questionnaire after reviewing the literature for research articles on falls among the elderly population, and the final version was reviewed by preventive medicine and family medicine consultants to ensure the clarity and validity of the questions. The questionnaire included four parts. The first part was about personal data and included questions about age, gender, nationality, marital status, education level, and monthly income. The second and third parts explored the prevalence and location of falls among participants in the prior three and 12 months, as well as the potential fall-related risk factors. The presence of depressive symptoms was assessed using the "Patient Health Questionnaire-2 (PHQ-2)". The last part is the 10-item Missouri Alliance for Home Care (MAHC-10) tool. It is a standardized, validated tool that explores 10 variables of fall risk. These include advanced age (>65 years), comorbidities of more than three medical diagnoses, history of falls within the last three months, incontinence, impaired vision, impaired functional mobility, polypharmacy, which refers to using four or more concurrent medications, cognitive impairment, chronic pain affecting the level of function (i.e., knee pain), and environmental hazards such as poor illumination, equipment tubing, inappropriate footwear, pets, hard-to-reach items, uneven or cluttered floor surfaces, and outdoor entry and exit points. Each item in this tool received a score of one if present and zero if absent. A total score of ≥4 indicated an increased risk of falling [23,24]. We performed an Arabic translation of the original English-language MAHC-10 tool, followed by a back-translation to English by two independent bilingual translators. Then, it was cross-checked with the original version to ensure the accuracy of the translation. The Arabic version of the risk assessment tool was pre-tested on a sample of the study population, and their results were not included in the final analysis.

Statistical analysis

All data were tabulated and analyzed by the statistical package for the social sciences software program, IBM SPSSStatistics for Windows, Version 26.0 (Released 2019; IBM Corp., Armonk, New York, United States). We presented categorical data as frequencies and percentages. We performed a univariate regression analysis to determine the risk factors significantly associated with falls in the preceding 12 months. This was followed by a multivariable regression analysis to determine a model of risk factors associated with falls in the preceding 12 months. Each variable that showed a p-value of 0.1 or less in the univariate analysis was included as an independent variable. Crude and adjusted odds ratios (OR) with 95% confidence intervals (CI) were presented. A p-value <0.05 was considered statistically significant.

## Results

The study included 296 participants. Approximately two-thirds were females (66.9%), aged 60-69 years old (68.2%), and married (68.9%). Most (93.9%) of them were Saudi. More than half (58.8%) were uneducated, and 40.2% were educated to the pre-university level. The monthly income varied between less than 5000 (68.9%) and more than 10000 (7.4%) Saudi Arabian Riyal (SAR) (Table 1).

**Table 1:** Sociodemographic characteristics of the study participants (N=296) N: number; SAR: Saudi Arabian Riyal

Variables		N=296	%
Gender	Female	198	66.9%
	Male	98	33.1%
Age, years	60 – 69	202	68.2%
	70 – 79	70	23.6%
	≥ 80 y	24	8.1%
Nationality	Saudi	278	93.9%
	Non-Saudi	18	6.1%
Marital status	Married	204	68.9%
	Single, divorced, widowed	92	31.1%
Education level	Uneducated	174	58.8%
	Pre-university	119	40.2%
	University	3	1.0%
Monthly income, SAR	< 5000	204	68.9%
	5000-10000	70	23.6%
	> 10000	22	7.4%

Obesity constituted 39.5%; comorbidities included gait impairment (52.7%), visual impairment (73.3%), memory loss (53.0%), urinary incontinence (57.8%), hypertension (67.2%), diabetes mellitus (DM) (66.6%), hyperlipidemia (50.7%), arthritis (70.6%), and transient ischemic attacks (31.4%). Furthermore, nearly one-third (30.7%) showed depressive symptoms, 37.2% of them reported using four or more concurrent drugs (polypharmacy), and 11.5% reported using sleeping pills. The commonly described environmental factors related to falls were inappropriate footwear (95.6%) and high door sills (71.3%), followed by uneven floor surfaces (45.9%) (Table 2).

**Table 2:** Distribution of the potential fall-related risk factors among the study participants (N=296) N: number; BMI: body mass index

Variables	N=296	%
BMI		
Normal	90	30.4%
Overweight	89	30.1%
Obesity	117	39.5%
Active elderly populations	205	69.3%
Regular physical exercise	18	6.1%
Smokers	79	26.7%
Comorbidities		
Gait impairment	156	52.7%
Visual impairment	217	73.3%
Memory loss	157	53.0%
Urinary incontinence	171	57.8%
Chronic disease	259	87.5%
Hypertension	199	67.2%
Diabetes mellitus	197	66.6%
Hyperlipidemia	150	50.7%
Arthritis	209	70.6%
Asthma	50	16.9%
Transient ischemic attacks	93	31.4%
Presence of depressive symptoms	91	30.7%
Use of sleeping pills	34	11.5%
Use of polypharmacy	110	37.2%
Environmental Factors		
Poor lighting	73	24.7%
Uneven ﬂoor surfaces	136	45.9%
Inappropriate footwear	283	95.6%
High door sills	211	71.3%

The self-reported prevalence of falls in the last three months was 14.5% (95% CI: 10.9-18.9) while the prevalence over the preceding 12 months was 25.3% (95% CI: 20.6-30.5%). Most falls over the last 12 months occurred indoors (73.3%), with the washroom (47.3%) being the most reported place. Regarding outdoor falls, streets (35.0%) followed by mosques (30.0%) were the most frequent locations of falls. Furthermore, the calculated risk score of falls revealed that a high percentage (72.3%) of the participants are at risk of falling (Table 3).

**Table 3:** Prevalence, location, and risk score of falls among the study participants (N=296) N: number; CI: confidence interval; *Risk of fall based on scores from the validated 10-item Missouri Alliance for Home Care fall risk assessment tool (scores of 4 or more indicate increased risk of fall)

Variables		N	%
History of falls (prevalence)	Last three months	43	14.5% (95% CI: 10.9-18.9)
	Last year	75	25.3% (95% CI: 20.6-30.5)
Location of falls over the last year	Indoor	55	73.3%
	Outdoor	20	26.7%
Location of indoor falls	Washroom	26	47.3%
	Kitchen	9	16.4%
	Hall	9	16.4%
	Front doorstep	5	9.1%
	Stairs	4	7.3%
	Bedroom	2	3.6%
Location of outdoor falls	Street	7	35.0%
	Masjid	6	30.0%
	Other	7	35.0%
Risk of fall*	No risk	82	27.7%
	At risk	214	72.3%

Risk factors that were significantly associated with falls during the preceding 12 months included gait impairment (OR: 2.543, 95% CI: 1.457-4.441, p<0.001), polypharmacy (OR: 1.834, 95% CI: 1.077-3.122, p=0.026), memory loss (OR: 1.827, 95% CI: 1.064-3.135, p=0.029), the presence of environmental factors (OR: 1.853, 95% CI: 1.091-3.144, p=0.022), and having risk of fall (OR: 2.165, 95% CI: 1.117-4.198) (Table 4).

**Table 4:** Univariate regression analysis for determining the risk factors of falls in the preceding 12 months *Significant at p<0.05; OR: odds ratio; CI: confidence interval; BMI: body mass index

Variables	Unadjusted OR (95% CI)	P-value
Gender (female)	0.792 (0.448-1.339)	0.422
Age group (70-79 years)	0.950 (0.505-1.786)	0.873
Age group (≥ 80 years)	1.219 (0.478-3.108)	0.678
Marital status (single, divorced, widowed)	0.974 (0.553-1.718)	0.928
Education (uneducated)	1.447 (0.839-2.496)	0.184
Income (<5000)	1.104 (0.387-3.145)	0.853
Income (5000-10000)	1.360 (0.442-4.185)	0.592
BMI (overweight)	1.081 (0.538-2.171)	0.827
BMI (obesity)	1.434 (0.758-2.712)	0.268
Activity	0.619 (0.357-1.072)	0.087
Absence of regular exercise	6.167 (0.806-47.153)	0.080
Smoking	0.910 (0.500-1.657)	0.759
Depressive symptoms	0.582 (0.317-1.070)	0.081
Use of sleeping pills	0.599 (0.238-1.509)	0.277
Polypharmacy	1.834 (1.077-3.122)	0.026*
Gait impairment	2.543 (1.457-4.441)	0.001*
Visual impairment	1.470 (0.787-2.747)	0.227
Memory loss	1.827 (1.064-3.135)	0.029*
Urine incontinence	1.416 (0.825-2.433)	0.207
Comorbidities	1.133 (0.671-1.915)	0.639
Environmental factors	1.853 (1.091-3.144)	0.022*
Risk of fall	2.165 (1.117-4.198)	0.022*

The multivariable analysis identified the presence of depressive symptoms, environmental factors, and gait impairment as significant independent predictors of fall occurrence. Older people with depressive symptoms were significantly associated with increased vulnerability to falls (AOR: 0.452, 95% CI: 0.239-0.854). Environmental factors were associated with 1.799 times (95% CI: 1.041-3.109) increased likelihood of fall. Gait impairment was the strongest risk factor (AOR: 2.775, 95% CI: 1.558-4.942) (Table 5).

**Table 5:** Multivariable regression analysis for determining the risk factors of falls in the preceding 12 months *Significant at p<0.05; AOR: adjusted odds ratio; CI: confidence interval

Variables	P-value	AOR	95% CI	Accuracy	P-value of the model
Depressive symptoms	0.014*	0.452	0.239-0.854	74.7%	<0.001*
Environmental factors	0.035*	1.799	1.041-3.109		
Gait impairment	0.001*	2.775	1.558-4.942		
Constant	0.000*				
Hosmer and Lemeshow test=0.542					

## Discussion

Falls are common among older adults and might lead to serious injuries, significantly impacting morbidity and mortality. The financial burden of falls on healthcare and social systems is also significant. Understanding the risk factors of falls might help establish evidence-based interventions and effective prevention strategies to reduce the frequency of falls [3,13,14,25].

Hence, this study was conducted among the elderly residents of Tabuk City, Saudi Arabia, to estimate the prevalence of falls and explore the various socio-demographic, individual, and environmental risk factors associated with them.

The present study showed that the prevalence of falls among the elderly in the preceding year was 25.3% (95% CI: 20.6-30.5%). The recorded prevalence rate is comparable to the reported WHO frequencies of falls among people aged 65 and older each year [26]. Previous research in Saudi Arabia revealed a higher prevalence rate of 31.5% in the Qassim region [15], and much higher prevalence rates in Jeddah (47.4%) [25], and in the Riyadh regions (49.9% and 57.7%) [13,14]. Corresponding research outside Saudi Arabia revealed conflicting results. Much lower rates have been reported from Japan (16.5%) [27] and the USA (22%) [7]. Studies from Ethiopia [28], UK [8], and Ecuador [29] have shown slightly higher rates of falls among the elderly (28%, 28%, and 30.3%, respectively). Studies from different regions of India detected a much higher prevalence of 36.6% [30] and 46.8% [19].

The observed disparity in the prevalence rates reported from different regions in Saudi Arabia might be attributed to differences in lifestyle or environmental conditions [15]. Differences in the characteristics of the studied populations, the prevalence of chronic illnesses, and the use of multiple drugs might explain the wide deviations between countries [25].

Falls among older adults result from complex individual factors and environmental circumstances. Generally, the prevalence of falls increases with the number of risk factors. The prevalence surges up to 78% among people with numerous risk factors [31].

In the current study, elderly individuals with gait impairment showed a significantly higher risk of falls (AOR: 2.775, 95% CI: 1.558-4.942). This finding agrees with Osoba et al. [32], who reported that age-related gait variability with abnormal gait patterns was a challenge for the elderly and was associated with an increased risk of falls due to balance problems. Additionally, Promsri et al. [33] concluded that increased walking instability was associated with an increased risk of falls, imposing training of the lower limbs to reduce the risk of falling. This finding is also in line with Alamri et al. [25], who revealed a significant association between lower-limb weaknesses, hip or knee replacement surgery, and the risk of falls. Gait disturbance in the elderly is related to different factors, such as sensory deficits and neurodegenerative diseases, including mild cognitive impairment (Alzheimer's disease), dementia, Parkinson’s disease, and vascular encephalopathy [34]. The management of the potential risk factors known to affect gait stability helps reduce the risk of falls among the elderly [35].

The present study also revealed that older people with depressive symptoms had significantly increased vulnerability to falls (AOR: 0.452, 95% CI: 0.239-0.854). In this context, several studies have reported that depressive symptoms characterized by sadness, loss of interest in activities, and reduced energy are consistently associated with the risk of falls and injuries among the elderly [36]. Hence, therapeutic interventions targeting reducing depressive symptoms would decrease the rate of falls.

Home settings and neighborhood environments have an impact on the individual health and well-being of older adults. In this study, the falls were significantly linked to a variety of environmental hazards, such as inappropriate footwear, poor lighting, clutter, uneven floors, and obstacles. This agrees with earlier reports [14,37] showing the influence of indoor environmental features on the incidence and severity of falls. Furthermore, several poor outdoor environmental conditions, such as uneven surfaces on streets, poor lighting, and traffic patterns, have also been reported as risk factors for falls [38]. Lee [39] found that indoor environmental hazards increased the risk of falls among women, while outdoor hazards mainly influenced falls among men. A recent longitudinal study explored the increased incidence of falls among the elderly, who reported a combination of housing and neighborhood problems and living alone after adjustment for individual factors. Living with others was protective and associated with no significant impact of environmental conditions on fall incidence [40].

Based on the calculated MAHC-10 fall risk assessment score, this study showed that participants at risk of falls had higher odds of falls (OR: 2.165, 95% CI: 1.117-4.198) in comparison to those who were not at risk. However, this relationship was not statistically significant in the multivariable regression model. Identification of older adults at risk of first or subsequent falls by family physicians or aged care providers certainly helps prevent such adverse events and the associated negative effects on health and quality of life [41].

Strengths and limitations

This study simultaneously investigated many intrinsic and extrinsic risk factors for falls, which may direct fall prevention programs. However, the cross-sectional survey design has an inherent recall bias and cannot establish causal relationships or analyze the impact of the identified risk factors on falls over time.

## Conclusions

The findings of the present study indicated that the prevalence of falls among the elderly residents of Tabuk City in the preceding year was 25.3%. The elderly at increased risk of falls include those with gait impairment, depressive symptoms, or who live in an inappropriate environmental condition, such as unfitting footwear, poor lighting, clutter, and uneven floors. Based on the identified risk factors, tailored interventions, including exercise, management of depression, and environmental monitoring, can effectively reduce the frequency of falls in high-risk communities. Primary healthcare providers play an important role in the prevention of falls among the elderly. Caregivers, along with the elderly, should be given more detailed health education related to fall prevention.
